# The Effect of Open and Closed Oocyte Vitrification Systems on Embryo Development: A Systematic Review and Network Meta-Analysis

**DOI:** 10.3390/jcm13092651

**Published:** 2024-04-30

**Authors:** Konstantinos Pantos, Evangelos Maziotis, Anna Trypidi, Sokratis Grigoriadis, Kristi Agapitou, Agni Pantou, Konstantinos Nikolettos, Georgia Kokkini, Konstantinos Sfakianoudis, Kimball O. Pomeroy, Mara Simopoulou

**Affiliations:** 1 Centre for Human Reproduction, Genesis Athens Clinic, Papanikoli, 15232 Athens, Greeceagni.pantou@genesisathens.gr (A.P.);; 2 Department of Physiology, Medical School, National and Kapodistrian University of Athens, 11527 Athens, Greece; 3 Obstetric-Gynecologic Clinic, Medical School, Democritus University of Thrace, 68100 Alexandroupolis, Greece; k.nikolettos@yahoo.gr; 4 Ivy Fertility, San Diego, CA 92127, USA

**Keywords:** oocyte vitrification, fertility preservation, open vitrification system, closed vitrification system, embryo development

## Abstract

**Background/Objectives**: Open and closed vitrification systems are commonly employed in oocyte cryopreservation; however, there is limited evidence regarding a comparison of their separate impact on oocyte competence. This study uniquely brings to the literature, data on the effect of open versus closed vitrification systems on laboratory and clinical outcomes, and the effect of cooling and warming rates. **Methods**: A systematic search of the literature was performed using the databases PubMed/MEDLINE and the Cochrane Central Library, limited to articles published in English up to January 2023. A network meta-analysis was conducted comparing each vitrification system versus fresh oocytes. **Results**: Twenty-three studies were included. When compared to fresh oocytes, both vitrification devices resulted in lower fertilization rates per MII oocyte retrieved. When comparing the two systems in terms of survival rates, no statistically significant difference was observed. However, interestingly open systems resulted in lower cleavage and blastocyst formation rates per 2 pronuclear (2PN) oocyte compared to fresh controls, while at the same time no statistically significant difference was detected when comparing closed devices with fresh oocytes. **Conclusions**: In conclusion, closed vitrification systems appear to exert a less detrimental impact on the oocytes’ competence, which is reflected in the blastocyst formation rates. Proof of superiority of one system versus the other may lead to standardization, helping to ultimately determine optimal practice in oocyte vitrification.

## 1. Introduction

Cryopreservation has been widely applied in relation to assisted reproductive technology (ART). Since the American Society for Reproductive Medicine (ASRM) lifted the experimental status of oocyte vitrification in 2013 [1], the employment of oocyte cryopreservation has increased over 10-fold in the past ten years [2]. Vitrification constitutes the gold standard method for oocyte cryopreservation, replacing slow freezing [3]. The fundamental principle of vitrification is ultrarapid cooling and warming with the use of increased concentrations of cryoprotectants [4], which increases the viscosity and increases the glass transition temperature, allowing vitrification at higher temperatures. This eliminates most ice formation during cooling, storage, and warming. The literature has demonstrated that vitrification may provide enhanced embryo quality and clinical outcomes compared to slow freezing [5,6]. 

Oocyte vitrification has been employed in fertility preservation, for cancer patients, where medication may induce ovarian damage, as well as in cases of elective fertility preservation to postpone childbearing [7]. Furthermore, vitrification of oocytes has resulted in the development and growth of oocyte donation programs and oocyte banking [8,9]. It has also been used to delay embryo transfer for medical reasons, or to increase the number of available oocytes for poor responders [7] via oocyte banking [10]. The wide applicability of oocyte vitrification warrants a solid consensus on embryo quality and outcomes. Current protocol variability includes variations in cryoprotectants [11], along with the option of open versus closed devices, and variations in temperatures and times for cryoprotectant exposure/removal. However, this lack of standardization may be responsible for inconsistencies, and the failure to identify optimal practices.

It has long been reported that oocyte vitrification leads to similar pregnancy rates between vitrified and fresh oocytes [12,13,14], and similar outcomes in oocyte donation programs [15,16]. Nevertheless, there are still concerns regarding the effectiveness of this method. According to a study investigating national data regarding clinical outcomes of vitrified and fresh donor oocytes, significantly higher live birth rates per cycle were reported when employing fresh oocytes [17] than vitrified and warmed oocytes. Further studies have reported a reduction in clinical outcomes with the employment of vitrified oocytes [18,19]. Regarding embryological outcomes, it has been reported that oocyte vitrification may impair fertilization rates [20] and embryo developmental potential [20,21]. The affected reproductive outcomes may be attributed to the following: a decreased number of embryos available for transfer, impaired morphokinetic parameters, altered DNA methylation patterns, and differential gene expression. The reduced number of surviving oocytes post-warming may decrease the number of oocytes available for insemination, as well as the number of blastocysts available for transfer [18]. Furthermore, it seems that embryo morphokinetic parameters [22,23,24], as well as the number and the diameter of nucleoli at the zygote stage [25], may be affected by the vitrification process. Additionally, increased levels of abnormal cleavage patterns, like direct cleavage and reverse cleavage, have been reported in regard to embryos obtained from vitrified oocytes [24]. During vitrification and warming, oocytes undergo temperature changes, osmotic stress, and cryoprotectant toxicity, as well as phase transitions. These conditions may affect oocyte quality, inducing oocyte spindle damage, ultrastructural changes, DNA damage, and epigenetic alterations [26]. It has been reported that oocyte vitrification may affect DNA methylation patterns in humans, as well as histone modifications and miRNA transcriptome in animal models [27]. Relative alterations have been investigated in gene expression. Particularly, the downregulation of several genes in the ubiquitination pathway has been established in human vitrified oocytes [27].

Proponents of open vitrification devices claim that the lower cooling and warming rates of closed vitrification devices may impair oocyte developmental potential. Those that opt for closed over open vitrification devices, point out that the decreased rates of cooling do not affect embryo development, while these devices offer similar warming rates and increased safety regarding possible contamination [28]. Vitrification systems can be classified as open and closed depending on whether there is direct contact of the oocytes with the liquid nitrogen. In open vitrification systems, ultrarapid freezing may be achieved; however, a potential increased risk of cross-contamination and disease transmission [29] has been described. Although viral cross-contamination and disease transmission between human oocytes through liquid nitrogen during cryostorage has not been established [30,31], the presence of environmental bacteria has been found in liquid nitrogen storage tanks [32]. No biological contamination was found in the open or closed devices stored in these tanks that contained environmental bacteria. In closed systems, the direct contact of oocytes with liquid nitrogen can be avoided, which may result in slower cooling or warming rates [3]. A decrease in survival rates has been reported in closed vitrification systems, possibly attributed to the slower cooling or warming rates [33,34]. However, no consensus can be reached in light of the conflicting contributing data [4].

Vitrification constitutes a simple, efficient, and cost-effective method, with high oocyte survival rates. Numerous studies have investigated the safety and the effectiveness of oocyte vitrification; however, the majority of them focus on conducting comparisons with the method of slow freezing. Additionally, the published data commonly report on the clinical outcomes, like pregnancy rates or live birth rates. Hitherto, there is lack of robust data regarding the impact of this method on oocyte competence and embryo developmental potential [26,35]. Furthermore, there is a limited number of studies investigating the effect of open and closed systems on the outcome of vitrification. It is timely and essential to elucidate the effect of oocyte vitrification, accounting for both open and closed systems, on embryo developmental potential. The fact that both systems are employed equally raises an important question pertaining to indicating which is superior. Achieving this may enable consistency in practice, which will in turn enable the standardization of processes and techniques in the ART laboratory, ascertaining optimal practice. This study aims to elucidate the effect of open and closed vitrification systems on oocyte competency, embryo development, and clinical data. This network meta-analysis evaluates this effect, employing both a direct and indirect comparison between the two vitrification systems. Further to this, meta-regression analysis allows the evaluation of the effect of cooling and warming rates on oocyte competency and embryo development. Our study aims to contribute towards achieving a consensus on evidence-based optimal practice in oocyte cryopreservation.

## 2. Materials and Methods

**Figure 1:**
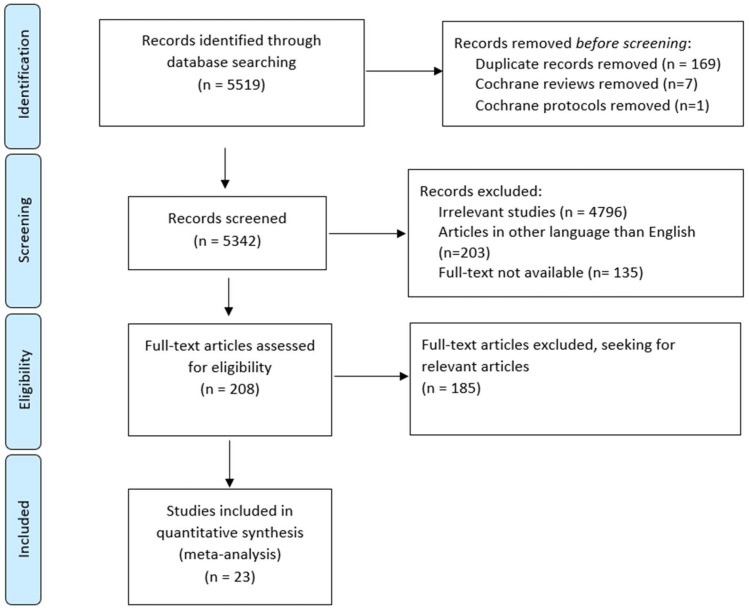
PRISMA flowchart. The PRISMA flowchart for the systematic review, detailing the search results.

A search of the electronic databases PubMed/MEDLINE and the Cochrane Central Library was conducted to retrieve studies published in English up to January 2023. During the search process, a sequence of key terms was used, along with their respective combinations: “IVF”, “ICSI”, “Blastocyst formation”, “Cleavage rate”, “Vitrification”, and “Cryopreservation”. The search strategy is provided in Supplementary Table S1. This broad search strategy ascertained the inclusion of all relevant studies. From the initial search, 5519 studies were retrieved from both PubMed/MEDLINE and the Cochrane Central Library databases. Specifically, 5154 studies were retrieved from the PubMed/MEDLINE database, and 365 from the Cochrane Central Library, of which 357 trials were found, which were included in the screening process of 7 reviews and 1 protocol, which were excluded as per the inclusion/exclusion criteria as outlined below. The initial screening of the titles and abstracts of all the studies led to the identification of 208 relevant published studies. This was followed by a full-text review of the studies, as well as a review of their bibliographic references, to identify additional relevant articles. The whole process was based on the PRISMA (‘Preferred Reporting Items for Systematic Reviews and Meta-Analyses’) diagram, which illustrates the flow of the search and the number of studies included or excluded at each stage (Figure 1). 

### 2.1. Inclusion/Exclusion Criteria

This meta-analysis consists of prospective studies conducted, employing human oocytes, and published in English. Literature reviews, case series, or reports were excluded. Retrospective studies were also excluded due to the increased risk of selection bias [36]. Abstract-only publications and studies in a language other than English were also excluded from this meta-analysis. The population was defined as women undergoing in vitro fertilization (IVF), employing an intracytoplasmic sperm injection (ICSI) with fresh oocytes, or oocytes derived from vitrification. The vitrified oocytes correspond to the study group, while the fresh oocytes correspond to the control group. The included studies investigated both the embryological outcomes, namely the fertilization rate, cleavage rate, blastocyst formation rate, and top-quality embryo rate, and the clinical outcomes, namely the clinical pregnancy rate, and the live birth rate. Regarding the various ovarian stimulation protocols employed per study, a subgroup analysis was not performed.

An initial study selection was performed by screening the study titles and abstracts to exclude studies that clearly did not meet the inclusion criteria. Following the initial screening, the full text of the remaining studies was obtained and thoroughly reviewed. A review of the references from the bibliography of all the relevant studies was performed as backward citation mining, followed by forward citation mining, employing Google Scholar. The study selection process was performed by four independent authors (AT, AP, KN, GK). Any disagreements between the authors were resolved by a senior author (MS). 

### 2.2. Risk of Bias

A risk of bias assessment was performed independently by two authors in regard to the studies included in this meta-analysis, employing the ROBINS-I tool [37]. Any disagreements were resolved by a third senior author.

### 2.3. Outcome Measures

The outcome measures in this study were the oocyte survival rate; fertilization rate; cleavage rate; top embryo quality rate; blastocyst formation rate; clinical pregnancy rate, as defined by the International Committee Monitoring Assisted Reproductive Technology (ICMART); and live birth rate. Two types of analysis were performed for the cleavage and blastocyst formation rate, namely the per 2 pronuclear (2PN) and per MII oocyte retrieved; the latter serving as an intention-to-treat analysis (ITT). 

### 2.4. Statistical Analysis

The network meta-analysis was performed, employing frequentist methods via the “netmeta” package in the R programming language for statistical purposes. A network meta-analysis is performed by comparing direct and indirect effects. The direct effect is estimated by the studies comparing the two different groups directly, while the indirect effect is estimated by comparing the two groups to another “reference” group. The reference group in the present study is the fresh oocytes group. A risk ratio with 95% confidence intervals was employed for the analyses of the included studies. Either the fixed effect or the random effects model was employed for results pooling, according to heterogeneity. The heterogeneity of the exposure effect was evaluated through the I^2^ statistic. An I^2^ value of 80% or greater indicated high heterogeneity and, thus, the meta-analysis was not performed. The random effects model was employed if the I^2^ value was greater than 0 and a significant sample size difference was observed between the studies, according to the 6th edition of the Cochrane Handbook. A chi-squared test for heterogeneity was also performed and the *p*-values were provided. Since the study sizes in this meta-analysis differed significantly, the fixed effects model was employed only if all the comparisons in a network presented with I^2^ = 0%. However, in all studies where high heterogeneity was observed, the random effects model was solely employed. To assess the efficiency of open and closed vitrification devices, the surface under the cumulative ranking curve (SUCRA) score was employed. The SUCRA score is a Bayesian metric, ranging from 0 to 1 (or 0% to 100%), to evaluate which treatment in a network meta-analysis is likely to be the most efficient [38]. Funnel plots for potential publication bias were conducted. The meta-regression analysis was performed in two arms, one evaluating the cooling and warming rates in studies comparing vitrified versus fresh oocytes, and the other evaluating the cooling and warming rate differences between open and closed vitrification. 

## 3. Results

**Figure 2:**
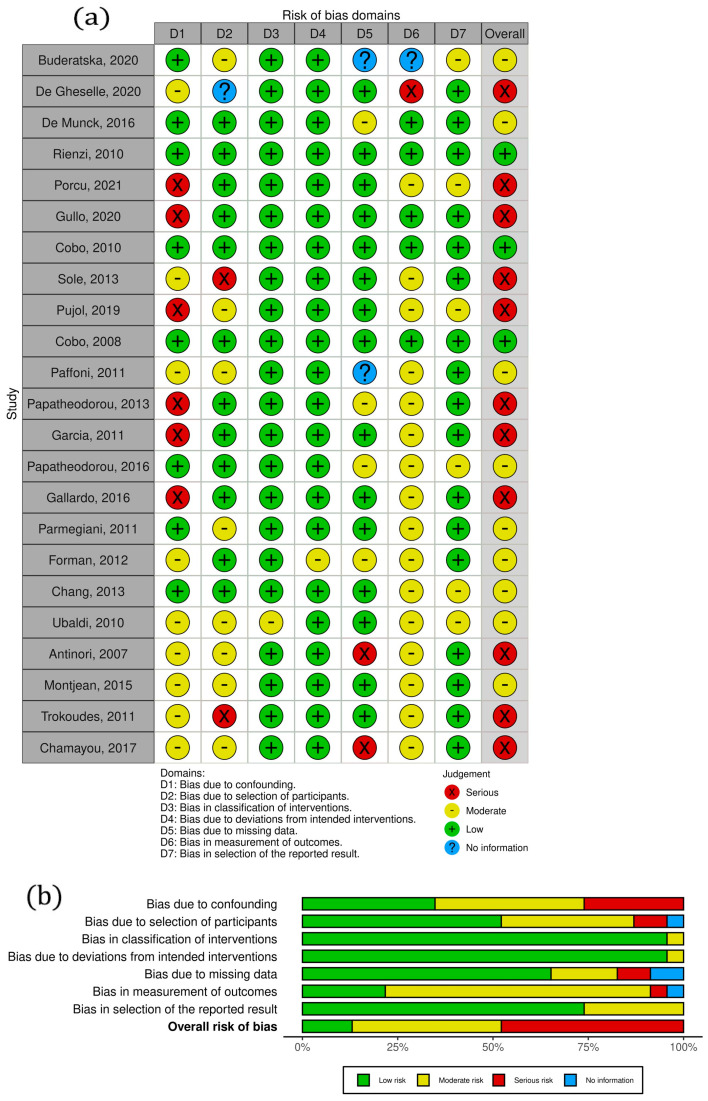
Risk of bias assessment: (**a**) “traffic light” plots of the domain-level judgements for each individual result, (**b**) weighted bar plots on the distribution of risk-of-bias judgements within each bias domain [4,10,13,14,15,23,33,34,39,40,41,42,43,44,45,46,47,48,49,50,51,52,53].

A total of 27,204 oocytes were included in the meta-analysis. Fifteen studies compared open vitrification systems with fresh oocytes, four studies compared the closed system with fresh oocytes, and five compared the two vitrification systems, closed vs. open. Detailed study characteristics are presented in Table 1. The bias assessment is presented in Figure 2. The treatment effects, along with their respective standard errors in regard to the fertilization rate, cleavage rate, blastocyst formation rate, and clinical pregnancy rate, are presented in Supplementary Tables S2–S7.

When comparing the survival of the oocytes, no statistically significant difference was observed between the two vitrification systems (RR: 1.00; 95% CI: 0.95–1.06), albeit with significant heterogeneity between the five studies (I^2^ = 88%). 

**Figure 3:**
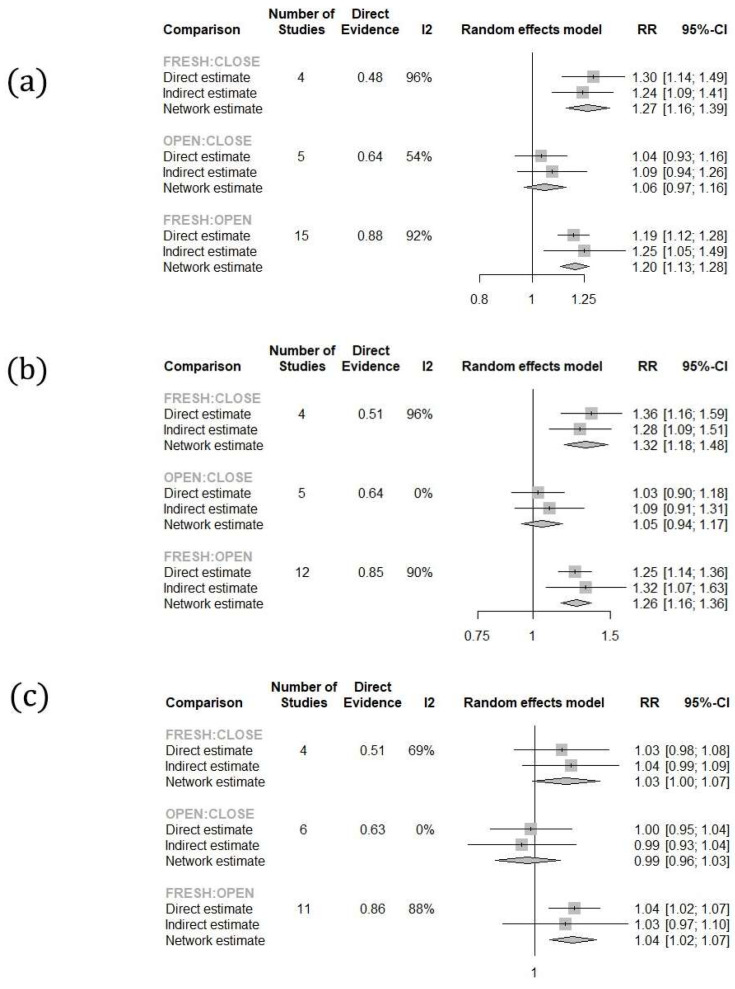
Forest plots of the results: (**a**) fertilization, (**b**) cleavage per MII, (**c**) cleavage per 2PN.

Comparing the fertilization rates per fresh and post-warming oocyte retrieved, both the open and closed vitrification systems presented with lower rates compared to fresh oocytes (Fresh vs. Open: RR: 1.20; 95% CI: 1.13–1.28; Fresh vs. Closed: RR: 1.27; 95% CI: 1.16–1.39). No statistically significant difference was observed between the two vitrification systems (Open vs. Closed: RR: 1.06; 95% CI: 0.97–1.16) (Figure 3). 

**Figure 4:**
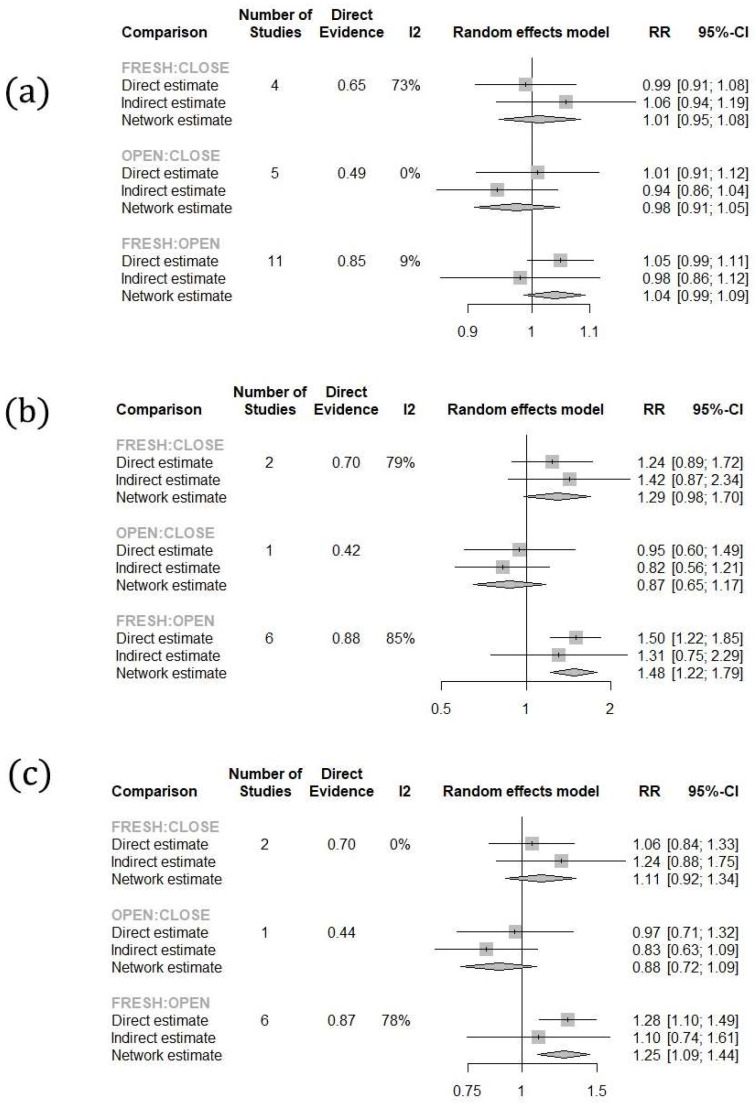
Forest plots of the results: (**a**) top quality embryos, (**b**) blastocyst formation per MII, (**c**) blastocyst formation per 2PN.

Similarly, regarding the cleavage rate per fresh and post-warming MII oocyte retrieved, both systems presented with lower rates compared to fresh oocytes (Fresh vs. Open: RR: 1.26; 95% CI: 1.16–1.36; Fresh vs. Closed: RR: 1.32; 95% CI: 1.18–1.48), while no statistically significant difference was observed between the two systems (RR: 1.05; 95% CI: 0.94–1.17). When evaluating the cleavage rates per 2PN zygote, open systems presented with lower rates compared to fresh oocytes (Fresh vs. Open RR: 1.04; 95% CI: 1.02–1.07), while marginally, a statistically significant difference was not established when comparing closed system vitrified and fresh oocytes (Fresh vs. Closed: RR: 1.03; 95% CI: 1.00–1.07). No statistically significant difference was observed between the two systems (RR: 0.99; 95% CI: 0.96–1.03) (Figure 3). No statistically significant difference was observed regarding the top quality embryo rate per 2PN zygote (Fresh vs. Open: RR: 1.04, 95% CI: 0.99–1.09; Fresh vs. Closed: RR: 1.01, 95% CI: 0.95–1.08; Open vs. Closed: RR: 0.98, 95% CI: 0.91–1.05) (Figure 4). 

When evaluating the blastocyst formation rate per fresh and post-warming MII oocyte retrieved, open systems presented with a significantly lower rate when compared to fresh oocytes (RR: 1.48; 95% CI: 1.22–1.79). No statistically significant difference was observed between closed vitrification systems and fresh oocytes, or open vitrification systems vs. closed (Fresh vs. Closed: RR: 1.29, 95% CI: 0.98–1.70; Open vs. Closed: RR: 0.87, 95% CI: 0.6–1.17). Similar results were observed when comparing the blastocyst formation rates per 2PN zygote (Fresh vs. Open: RR: 1.25, 95% CI: 1.09–1.44; Fresh vs. Closed: RR: 1.11, 95% CI: 0.92–1.34; Open vs. Closed: RR: 0.88, 95% CI: 0.72–1.09) (Figure 4). Employing the SUCRA score to rank the treatments, fresh oocytes presented with the best score of 0.9255 for blastocyst formation, followed by closed vitrification systems (SUCRA score: 0.5175) and open vitrification systems (SUCRA score: 0.0570). No statistically significant difference was observed regarding clinical pregnancy (Fresh vs. Open: RR: 1.03, 95% CI: 0.91–1.15; Fresh vs. Close: RR: 1.09, 95% CI: 0.94–1.26; Closed vs. Open: RR: 1.06, 95% CI: 0.93–1.22), and live birth rates (Fresh vs. Open: RR: 1.04, 95% CI: 0.84–1.29; Fresh vs. Close: RR: 1.04, 95% CI: 0.84–1.30; Closed vs. Open: RR: 1.01, 95% CI: 0.82–1.24). To provide a clear level of evidence for each outcome, a GRADE (Grading of Recommendations, Assessment, Development, and Evaluations) assessment was performed (Table 2).

A meta-regression analysis was performed to evaluate the effect of cooling and warming rates. The meta-regression analysis was performed in two arms, one evaluating the cooling and warming rates in studies comparing vitrified versus fresh oocytes, and the other evaluating the cooling and warming rate differences between open and closed vitrification systems. In the first arm of the study, warming rates appear to be positively associated with the fertilization rate (*p* = 0.03). Moreover, warming rates were positively associated with a marginally non-statistically significant association with the cleavage rate (*p* = 0.052). No significant association was observed regarding the blastocyst formation rates. When comparing the cooling and warming rate differences, in studies comparing open and closed vitrification systems, only warming rates were positively associated with the fertilization rate (*p* = 0.01). No other statistically significant association was observed.

## 4. Discussion

Oocyte cryopreservation has achieved routine clinical practice status, both for medical and social reasons. The advent of vitrification improved oocyte cryopreservation and resulted in providing a real option for fertility preservation for social reasons. Vitrification allowed for more effective cryopreservation for both medical and social reasons, enhancing reproductive autonomy and protecting the reproductive rights of women and, thus, its usefulness cannot be debated. It has been used extensively for both oocyte and embryo donation, as well as by cryobanks. While it may seem early to provide robust results on the utilization of oocytes following cryopreservation for any of the above mentioned reasons, the preliminary data seems to be encouraging [54]. As cryopreservation is viewed as a service, consistency and reported clinical outcomes are of upmost importance, rendering this meta-analysis timely and essential. This served as an incentive for this study. Our meta-analysis was divided into two arms. The first arm compared the clinical outcomes of fresh versus vitrified oocytes, and the second between open and closed device systems. It is certain that cryopreservation practice will keep evolving in ART and its use will continue to be extended, despite some reported discouraging results [55]. Nonetheless, future implementation should be shaped by data on efficiency and safety, leading to standardization, consistency, and optimal practice. The considerable variation in practice involving closed and open vitrification systems adds another level of complexity when contemplating optimal practice and renders a comparison and respective associations in embryological and clinical outcomes imperative. This served as an incentive for the second arm of the study, comparing the two vitrification systems, to assess their safety and efficiency, in order to draw conclusions toward optimal clinical practice.

According to the findings of our study, when comparing open and closed vitrification systems, similar results were observed for all outcome measures assessed. When comparing vitrified with fresh oocytes, both open and closed vitrification systems presented with a lower fertilization rate per MII oocyte retrieved, as well as a lower cleavage and top quality embryo rate, both per 2PN and per MII oocyte vitrified. We found no statistical differences in the blastocyst formation rates when comparing closed to fresh or open systems. However, open vitrification systems resulted in lower blastocyst formation rates when compared to fresh oocytes. While this result regarding the blastocyst formation rates may seem confusing, it is supported by the SUCRA score. According to the SUCRA score, which provides a ranking of the treatment efficiency, fresh oocytes seem to present with optimal results, followed by closed vitrification systems, while the open system ranks last. When examining the clinical pregnancy rate and live birth rate, no statistically significant difference was observed, as well as between the two vitrification systems, open and closed. The lower blastocyst formation rate, as well as the lower cleavage rate indicates a higher developmental arrest rate. Albeit, an outcome of developmental arrest may be attributed to a number of factors, ranging from intrinsic cultural conditions to different techniques employed, it appears that the choice of open versus closed vitrification may be implicated as a causative factor in this phenomenon.

Our data indicate that the open system could exert a negative effect, extending to the dynamic of the oocyte to subsequently form blastocysts. However, the authors refrain from making bold statements, as basic research data to provide an adequate explanation are lacking. A focal difference between open and closed vitrification systems is that during open vitrification the oocytes are in direct contact with liquid nitrogen, whereas during closed vitrification the oocytes are sealed in carriers. More data focusing on the impact of this aspect, analyzing the evidence from a cryobiology perspective, are needed. What is more, the lack of direct contact with liquid nitrogen in closed systems lowers the cooling rate of oocytes, ranging from −522 to −1220 °C per minute, when compared to −15,000 °C per minute in open systems, identifying another focal difference [56,57]. Warming rates are also potentially affected when using a closed device versus an open one.

Vitrification is a technique constantly subject to improvement efforts, from introducing new carrier devices to different protocols for cooling or warming. This dynamic process of improving the systems’ efficiency renders a cross-sectional comparison between open and closed systems subject to relative compromise, as the respective systems are modified over time. It should be noted that closed vitrification devices have improved their cooling rate over the years, and this may present a reason for caution when comparing the two methods from a cross-sectional perspective, as the year of study may imply that a different cooling rate was achieved. Efforts in improving the cooling or warming rates have always been at the top of the agenda when aiming to improve vitrification results. Nonetheless, it may be postulated that achieving a cooling rate above a specific cut-off point may correspond to a plateau in improvement, above which the results may not be further improved [4]. On the matter of debating whether cooling or warming rates appear to be more important and impactful, highly cited studies have been contributed, albeit mostly investigating animal models. In fact, warming rates appear to be more important than cooling rates in a study demonstrating that ultra-rapid warming rates result in adequate oocyte survival, even with low cooling rate of −880 °C/min [58]. According to the results of our meta-regression analysis, it seems that only warming rates affect fertilization rates. While no significant association was observed with the cleavage and blastocyst formation rates, this may be attributed to the limited sample size. This association highlights the importance of the warming rate, which seems to influence oocyte potential to a greater extent compared to cooling rates. Our meta-analysis data on humans, supported by animal model studies, on the superiority of the warming rate value should fuel respective basic research. In the field of vitrification as a whole, a considerable number of studies have been dedicated to improving results; nonetheless, this effort has not been buttressed and paired adequately with basic research studies. This ever-evolving field could benefit from more studies on cryobiology and the effect of vitrification and its various protocols on the transcriptomic and translational profile of oocytes and embryos. Such contributions could provide evidence on the biological aspects entailed, explaining why the open system could exert a more negative impact, negatively influencing the biological dynamic of the vitrified oocyte. 

The effect of vitrification on oocyte and embryo potential could be mediated through alterations in the DNA methylation patterns. It is generally believed that the DNA methylation status of oocytes and early embryos is highly sensitive to external stimuli, which may potentially lead to poor developmental capacity and embryo quality. Vitrification may act as the stimulus that affects the DNA methylation pattern of oocytes [27]. Further studies investigating genome-wide methylation patterns of human cryopreserved MII oocytes are necessary to elucidate the effect of vitrification. Further to this, vitrification leads to differential expression of several genes involved in a number of processes, namely ubiquitination and autophagy, cell cycle regulation, DNA repair, and metabolic and mitochondrial pathways [27,59,60,61]. Studies have reported that vitrification does not modify the expression of genes essential for oocyte development and cytokinesis [62,63]. Conflicting data should be delineated to gain an in-depth understanding. 

Despite a seeming abundance of data on vitrification, it appears that the right studies with respect to design and sample size are still needed to draw robust conclusions. The findings in this study fuel the need for further data, particularly focusing on the effects of direct contact with liquid nitrogen on embryo physiology and dynamics. It is possible that the scientific community may need to consider that the survival of oocytes, as described herein by all studies, may not be fit to serve as a reliable indicator. The outcome measure of oocyte survival is commonly encountered in the literature and the scientific community would benefit from understanding the definition of oocyte survival and whether it is different to the sustenance of the oocyte’s original morphology. The physiology of the oocyte is complex and any detrimental impact on it will not be indicated purely by a morphological observation. Therefore, oocyte survival, as a commonly used outcome measure in research studies, may be of limited significance. Reporting on oocyte survival may certainly be of value as a first indicator of success; however, it should not be employed as the sole metric when assessing oocyte cryopreservation. Even though there have been reports on oocyte survival in multiple attempts at re-vitrification [64], this data may well raise the point concerning the need to study in-depth the effect of direct contact. Further to this, embryo re-vitrification has been shown to result in lower live birth rates and higher miscarriage rates [65]. This may be attributed to different expression levels of mir-16 and mir-let-7a, inducing an epigenetic effect on blastocysts [66]. Thus, it is of upmost importance to evaluate the indicators that will clarify the safety and the efficacy of oocyte and embryo re-vitrification. 

This network meta-analysis poses intrinsic strengths and limitations. In this type of analysis, both direct and indirect evidence are estimated in a network. This enables a more robust analysis, even in cases with lower sample sizes. As open and closed vitrification types have been directly compared and separately compared with fresh oocytes, all nodes in the network are directly connected, thus enhancing the robustness of the results. Despite this, the different study designs may be regarded as an intrinsic limitation of the study. Furthermore, the heterogenous number of studies included in a number of outcomes may present a significant limitation. It should be mentioned that only two studies reported results on the blastocyst formation rate, comparing closed vitrification systems to fresh oocytes, and only one study comparing open and closed vitrification systems on this outcome. This lack of studies leads to weak evidence when interpreting the blastocyst formation rate outcome. Further to this, in each type of comparison, different protocols regarding the method of vitrification were employed, posing as another limitation.

The small sample size when reporting on the blastocyst formation, clinical pregnancy, and live birth rates is a limitation in the present study. Moreover, the high heterogeneity observed may present another reason for caution when interpreting the results of this study. Furthermore, the significant heterogeneity in the controlled ovarian stimulation (COS) protocols employed per study may constitute another reason for caution. The enrolled studies described different protocols featuring different doses and durations for ovarian stimulation, employing the administration of different gonadotropin-releasing hormone (GnRH) analogues in combination with a follicle-stimulating hormone (FSH) or human menopausal gonadotropin (hMG). Moreover, for ovulation induction, human chorionic gonadotropin (hCG) or a GnRH agonist was employed. Subgroup analysis, based on COS protocols, was not performed in this network meta-analysis, due to the limited number of studies that provide data regarding the number of patients that were subjected to each protocol. It may be possible that the different COS protocols employed may have exerted an effect on both the laboratory outcomes, as well as the clinical pregnancy outcomes. Variations in the vitrification devices employed may be another identified limitation, herein. Regarding the open vitrification systems, the most commonly employed device was the Cryotop method, while no device was indicated as more commonly employed in the closed system. The significant percentage of studies evaluated as of high or moderate overall risk of bias, mainly attributed to bias due to confounding, presents as another limitation of this meta-analysis. A limitation of the meta-regression analysis on cooling versus warming rates, is the fact that it was performed in two arms. However, this was deemed necessary, as according to our knowledge a method that would enable the simultaneous comparison of the cooling and warming rates and their difference between open and closed systems, including all possible limitations of this comparison, has not yet been developed. On another note, it should be mentioned that all studies included in this meta-analysis employed dimethyl sulfoxide (DMSO) in regard to the vitrification protocols. It could be of interest to evaluate the effect of DMSO-free protocols; however, further studies are required. The fact that the top quality embryo, based on morphology, is a subjective evaluation constitutes an intrinsic limitation in regard to this outcome of the network meta-analysis. Time lapse is making its way toward becoming a requirement in IVF laboratories; however, we still have some way to go to reach this status. In the meantime, it is undeniable that time lapse has long been considered an excellent research tool in clinical embryology studies. It is possible that future studies may provide more morphokinetic data, leading to more conclusive data on the matter of the impact of vitrification on oocyte competency and embryo development. In addition to this, artificial intelligence (AI) may assist in objectively evaluating embryos and, thus, is a valuable tool in such research. This meta-analysis is mainly clinically oriented, evaluating laboratory and clinical outcomes. Nonetheless, it is important for future studies to elucidate the biological effects of vitrification and its mechanism of action in order to arrive at improved clinical outcomes. 

Both open and closed oocyte vitrification systems seem to impact oocyte competence and subsequent embryo development. The choice of open or closed vitrification systems seems to be up to “the consumer’s discretion”, and subject to marketing and other promotions. However, in the post-pandemic corona virus disease (COVID-19) period, it has been highlighted that use of closed systems offer the safety of protection from the potential of cross-contamination [50]. The well-reported advantage of the closed system in protecting against cross-contamination and disease transmission may serve as an additional incentive toward closed system use. The abundance of indications and extensive employment of vitrification warrants ascertainment of the safety of the procedure. It has been recently reported that closed vitrification systems offer enhanced safety when compared to open ones [67]. Whether this outweighs the open device’s simplicity and increased cooling rates by avoiding the insulative properties of closed systems, remains to be seen. Reaching a consensus on open versus closed vitrification systems is timely and essential. This data raises the point that, albeit performing equally, the closed system is associated with better blastocyst formation rates. The proof of superiority of one system versus the other may lead to standardization, helping to ultimately determine optimal practice. In the era of precision medicine, and in light of a recent study associating cryopreservation and offspring cancer [55], reporting on the safety and effectiveness of the use of cryopreservation on the health of children and future adults originating from cryopreserved oocytes is of upmost importance. 

## 5. Conclusions

The outcome measures of interest when considering cryopreservation are currently shifting from clinical pregnancy and live birth rates to neonatal outcomes and to the health of offspring in adulthood. Thus, the scientific community must ensure safety and efficacy through standardization and consistency. To achieve this, further large randomized controlled trials (RCTs) and longitudinal studies are required. The data presented herein, supports that when comparing the two systems (open and closed), they seem to perform equally in terms of oocyte competence and embryo development. However, when comparing the two systems indirectly with fresh oocytes, closed systems seem to exert a less detrimental impact on oocytes’ competence, reflected in higher blastocyst formation rates. Concurrently, even though there is no data indicating that cross-contamination from one device to another occurs during storage, the use of closed devices to prevent contamination with environmentally contaminated liquid nitrogen may warrant the consideration of using a closed device. Therefore, taking into account the improved blastocyst formation rates noted herein, when the closed system is used, coupled by the fact that the closed system ascertains an added level of safety, it appears that closed systems offer an additional advantage. Further studies, and particularly RCTs comparing the two systems, are required prior to cement our findings, while basic research regarding the effect of liquid nitrogen’s direct contact on the oocyte’s physiology is pending. Basic research should be conducted to buttress, and ultimately explain, the data from clinical studies. 

## Figures and Tables

**Table 1:** Summary of the characteristics of the studies included. Description of design, comparison, population, number of cycles, vitrification method, number of MII oocytes, and outcomes, reported per study. BF = blastocyst formation, CP = clinical pregnancy, LB = live birth.

	Design	Comparison	Patients	Number of Cycles	Vitrification Method	Number of MII Oocytes	Outcomes
[39]	Prospective	Fresh vs. open	Autologous	251 cycles	Cryotop	330 open system, 726 fresh oocytes	Fertilization, cleavage rate
[40]	Prospective	Fresh vs. open	Donors	29 donor cycles	Cryotech	121 open system, 262 fresh oocytes	Fertilization, cleavage rate, top quality embryos, BF, top quality blastocysts
[41]	Prospective	Fresh vs. open	Autologous	22 cycles	Cryotop	186 open system, 204 fresh oocytes	Fertilization, top quality embryos, BF
[42]	Prospective	Fresh vs. open	Autologous	69 cycles	Cryotop	615 open system, 463 fresh oocytes	Fertilization, BF
[10]	RCT	Fresh vs. open	Donors	584 donor cycles, 600 recipient cycles (300 open system, 300 fresh)	Cryotop	3286 open system, 3185 fresh oocytes	Fertilization, cleavage rate, top quality embryos, CP, LB
[15]	RCT	Fresh vs. open	Donors	30 donor cycles, 30 recipient cycles	Cryotop	231 open system, 219 fresh oocytes	Fertilization, cleavage rate, top quality embryos
[23]	Prospective	Fresh vs. open	Donors	27 donor cycles, 67 recipient cycles (36 fresh, 31 open system)	Cryotop^®^	287 open system, 220 fresh oocytes	Fertilization, cleavage rate, top quality embryos, BF, top quality blastocysts, LB
[43]	RCT	Open vs. closed	Donors	42 donor cycles, 78 recipient cycles	CryotopSC High Security CBSvit	257 open system, 253 close system	Fertilization, cleavage rate, top quality embryos
[44]	RCT	Fresh vs. open	Autologous	44 cycles	Cryotop	294 open system, 294 fresh oocytes	Fertilization, cleavage rate, BF
[45]	Prospective	Fresh vs. closed	Donors	14 donor cycles, 14 recipient cycles	SafeSpeed	68 close system, 75 fresh oocytes	Fertilization, cleavage rate, top quality embryos
[46]	Prospective	Fresh vs. closed	Donors	78 donor cycles (20 close system, 58 fresh), 119 recipient cycles (34 close system, 85 fresh)	Cryolock	283 close system, 696 fresh oocytes	Fertilization, cleavage rate, top quality embryos, BF, top quality blastocysts, CP, miscarriage
[47]	RCT	Open vs. closed	Donors	97 donor cycles, 190 recipient cycles (95 open system, 95 close system)	VitriSafe, Cryotop^®^	784 close system, 790 open system	Fertilization, cleavage rate, top quality embryos, BF, top quality blastocysts, CP, LB
[48]	Prospective	Fresh vs. open	Autologous	90 cycles	Cryotop	684 open system, 540 fresh oocytes	Fertilization, cleavage rate, BF
[33]	Prospective	Fresh vs. open	Autologous	102 cycles (53 open system, 49 fresh)	Cryotop	268 open system, 130 fresh oocytes	Fertilization, cleavage rate, top quality embryos
[33]	Prospective	Fresh vs. closed	Autologous	99 cycles (51 close, 48 fresh)	Cryotip	261 close system, 135 fresh oocytes	Fertilization, cleavage rate, top quality embryos
[34]	RCT	Open vs. closed	Donors	78 donor cycles, 150 recipient cycles (75 open system, 75 close system)	Vitrisafe	598 close system, 608 open system	Fertilization, cleavage rate, top quality embryos, CP, miscarriage, LB
[49]	Prospective	Fresh vs. closed	Donors	92 donor cycles, 184 recipient cycles (92 close system, 92 fresh)	Vitrisafe	984 close system, 982 fresh oocytes	Fertilization, cleavage rate, top quality embryos, BF, top quality blastocysts, CP, miscarriage, LB
[13]	RCT	Fresh vs. open	Autologous	31 cycles	Cryotop	168 open system, 120 fresh oocytes	Fertilization, cleavage rate, top quality embryos
[50]	RCT	Open vs. closed	Autologous	737 cycles (368 close system, 369 open system)	Cryotop^®^, High-Security Vitrification™	1469 close system, 1095 open system	Fertilization, cleavage rate, top quality embryos, CP, miscarriage
[4]	Prospective	Open vs. closed	Donors	83 donor cycles, 80 recipient cycles (40 open system, 40 close system)	Rapid-i^®^, Cryotop^®^	498 close system, 474 open system	Fertilization, cleavage rate, top quality embryos, CP, miscarriage, LB
[14]	RCT	Fresh vs. open	Autologous	40 cycles	Cryotop	124 open system, 120 fresh oocytes	Fertilization, cleavage rate, top quality embryos
[51]	Prospective	Fresh vs. open	Donors	99 donor cycles, 198 recipient cycles (99 open system, 99 fresh)	Cryotop	990 open system, 1099 fresh oocytes	Fertilization, cleavage rate, top quality embryos, CP, miscarriage, LB
[52]	Prospective	Fresh vs. open	Donors	36 donor cycles, 77 recipient cycles (36 open system, 41 fresh)	Cryotop	210 open system, 247 fresh oocytes	Fertilization, cleavage rate, top quality embryos, CP, LB
[53]	Prospective longitudinal	Fresh vs. open	Autologous	182 cycles	Cryotop	770 open system, 537 fresh oocytes	Fertilization, top quality embryos

**Table 2:** GRADE assessment on the level of certainty of the body of evidence for each outcome. ⨁⨁◯◯ Low: Further research is very likely to have an important impact on our confidence in the estimate of effect and is likely to change the estimate. ⨁◯◯◯ Very low: Any estimate of effect is very uncertain.

	Number of Studies	RR (95% CI)	Certainty
Fertilization			
Fresh vs. Closed	4	1.27 (1.16–1.39)	⨁⨁◯◯ LOW
Open vs. Closed	5	1.06 (0.97–1.16)	
Fresh vs. Open	15	1.20 (1.13–1.28)	
Cleavage per MII			
Fresh vs. Closed	4	1.32 (1.18–1.48)	⨁⨁◯◯ LOW
Open vs. Closed	5	1.05 (0.94–1.17)	
Fresh vs. Open	12	1.26 (1.16–1.36)	
Cleavage per 2PN			
Fresh vs. Closed	4	1.03 (1.00–1.07)	⨁⨁◯◯ LOW
Open vs. Closed	6	0.99 (0.96–1.03)	
Fresh vs. Open	11	1.04 (1.02–1.07)	
Top quality embryo			
Fresh vs. Closed	4	1.01 (0.95–1.08)	⨁⨁◯◯ LOW
Open vs. Closed	5	0.98 (0.91–1.05)	
Fresh vs. Open	11	1.04 (0.99–1.09)	
Blastocyst formation per MII			
Fresh vs. Closed	2	1.29 (0.98–1.70)	⨁◯◯◯ VERY LOW
Open vs. Closed	1	0.87 (0.6–1.17)	
Fresh vs. Open	6	1.48 (1.22–1.79)	
Blastocyst per 2PN			
Fresh vs. Closed	2	1.11 (0.92–1.34)	⨁◯◯◯ VERY LOW
Open vs. Closed	1	0.88 (0.72–1.09)	
Fresh vs. Open	6	1.25 (1.09–1.44)	
Clinical pregnancy			
Fresh vs. Closed	2	1.09 (0.94–1.26)	⨁◯◯◯ VERY LOW
Closed vs. Open	5	1.06 (0.93–1.22)	
Fresh vs. Open	4	1.03 (0.91–1.15)	
Live birth			
Fresh vs. Closed	1	1.04 (0.84–1.30)	⨁◯◯◯ VERY LOW
Closed vs. Open	3	1.01 (0.82–1.24)	
Fresh vs. Open	3	1.04 (0.84–1.29)	

## Data Availability

The data underlying this article will be shared on reasonable request to the corresponding author.

## Supplementary Materials

The following supporting information can be downloaded at: https://www.mdpi.com/article/10.3390/jcm13092651/s1, Table S1: Search strategy; Table S2: Treatment effect and standard error per study for the fertilization rate per oocyte retrieved outcome; Table S3: Treatment effect and standard error per study for the cleavage rate per oocyte retrieved outcome; Table S4: Treatment effect and standard error per study for the cleavage rate per 2PN zygote outcome; Table S5: Treatment effect and standard error per study for the blastocyst formation rate per oocyte retrieved outcome; Table S6: Treatment effect and standard error per study for the blastocyst formation rate per 2PN zygote outcome; Table S7: Treatment effect and standard error per study for the clinical pregnancy rate outcome.
