# Supplementary material for: The Effect of Open and Closed Oocyte Vitrification Systems on Embryo Development: A Systematic Review and Network Meta-Analysis
Source: J Clin Med. 2024 Apr 30;13(9):2651. doi: 10.3390/jcm13092651 (PMC11084263; doi:10.3390/jcm13092651)
Supplement: Supplementary file 1 [file jcm-13-02651-s001.zip › jcm-2942729-supplementary.pdf]

Supplementary Table S1: Search strategy

Database: Pubmed/ MEDLINE

(((IVF) OR (ICSI) OR (Vitrification) OR (Cryopreservation)) AND ((Blastocyst formation) OR (Cleavage rate))) (n=5.154)

Query	Search Details	Results
(((IVF) OR (ICSI) OR (Vitrification) OR (Cryopreservation)) AND ((Blastocyst formation) OR (Cleavage rate)))	("j in vitro fert embryo transf"[Journal] OR "ivf"[All Fields] OR ("sperm injections, intracytoplasmic"[MeSH Terms] OR ("sperm"[All Fields] AND "injections"[All Fields] AND "intracytoplasmic"[All Fields]) OR "intracytoplasmic sperm injections"[All Fields] OR "icsi"[All Fields]) OR ("vitrificated"[All Fields] OR "vitrification"[MeSH Terms] OR "vitrification"[All Fields]) OR ("cryopreservability"[All Fields] OR "cryopreservable"[All Fields] OR "cryopreservant"[All Fields] OR "cryopreservants"[All Fields] OR "cryopreservated"[All Fields] OR "cryopreservation"[MeSH Terms] OR "cryopreservation"[All Fields] OR "cryopreserved"[All Fields] OR "cryopreservations"[All Fields] OR "cryopreservative"[All Fields] OR "cryopreservatives"[All Fields] OR "cryopreserve"[All Fields] OR "cryopreserving"[All Fields])) AND ((("blastocyst"[MeSH Terms] OR "blastocyst"[All Fields] OR "blastocysts"[All Fields]) AND ("formations"[All Fields] OR "metabolism"[MeSH Terms] OR "metabolism"[All Fields] OR "formation"[All Fields])) OR (("cleavaged"[All Fields] OR "cleavages"[All Fields] OR "cytokinesis"[MeSH Terms] OR "cytokinesis"[All Fields] OR "cleavage"[All Fields]) AND ("j rehabil assist technol eng"[Journal] OR "rate"[All Fields])))	5.154

Database: Cochrane Library

(((IVF) OR (ICSI) OR (Vitrification) OR (Cryopreservation)) AND ((Blastocyst formation) OR (Cleavage rate))) (n=365)

Supplementary Table S2: Treatment effect and standard error per study for the fertilization rate per oocyte retrieved outcome.

AUTHOR	YEAR	Treatment Effect	Standard Error of Treatment Effect	Group 1	Group 2	2PN zygotes group 1	MII oocytes retrieved group 1	2PN zygotes group 2	MII oocytes retrieved group 2
De Munck	2016	0.050271	0.05619	OPEN	CLOSE	188	257	176	253

Rienzi	2010	-0.08408	0.064253	OPEN	FRESH	95	124	100	120
Porcu	2021	0.062181	0.049353	OPEN	CLOSE	445	1095	561	1469
Gullo	2020	-0.01934	0.028391	OPEN	CLOSE	594	790	601	784
Cobo	2010	-0.06521	0.015919	OPEN	FRESH	2256	3286	2334	3185
Sole	2013	-0.18361	0.026667	OPEN	FRESH	665	990	887	1099
Pujol	2019	0.138261	0.054782	OPEN	CLOSE	294	474	269	498
Buderatska	2020	0.013032	0.048061	OPEN	FRESH	102	121	218	262
De Gheselle	2020	-0.25328	0.067449	OPEN	FRESH	160	287	158	220
Cobo	2008	-0.10464	0.050083	OPEN	FRESH	171	231	180	219
Paffoni a	2011	-0.56498	0.085429	OPEN	FRESH	116	268	99	130
Paffoni b	2011	-0.85678	0.098435	CLOSE	FRESH	87	261	106	135
Papatheodorou	2013	-0.02395	0.039888	OPEN	CLOSE	406	608	409	598
Garcia	2011	-0.258	0.043703	CLOSE	FRESH	191	283	608	696
Trokoudes	2011	-0.1161	0.045114	OPEN	FRESH	162	210	214	247
Chamayou	2017	-0.10303	0.039531	OPEN	FRESH	411	615	343	463
Papatheodorou	2016	-0.06959	0.023786	CLOSE	FRESH	744	984	796	982
Gallardo	2016	0.004454	0.097086	CLOSE	FRESH	51	68	56	75
Parmegiani	2011	-0.32708	0.067038	OPEN	FRESH	107	168	106	120
Forman	2012	-0.35239	0.048003	OPEN	FRESH	187	294	266	294
Chang	2013	-0.11778	0.065743	OPEN	FRESH	124	186	153	204
Antinori	2007	-0.04516	0.017189	OPEN	FRESH	305	330	702	726
Montjean D	2015	-0.11352	0.045973	OPEN	FRESH	376	648	351	540
Ubaldi	2010	-0.3733	0.033769	OPEN	FRESH	462	770	468	537

Supplementary Table S3: Treatment effect and standard error per study for the cleavage rate per oocyte retrieved outcome.

AUTHOR	YEAR	Treatment Effect	Standard Error of Treatment Effect	Group 1	Group 2	Cleaved embryos group 1	MII oocytes retrieved group 1	Cleaved embryos group 2	MII oocytes retrieved group 1

De Munck	2016	0.112489	0.090676	OPEN	CLOSE	133	257	117	253
Rienzi	2010	-0.10536	0.065991	OPEN	FRESH	93	124	100	120
Porcu	2021	0.059798	0.053498	OPEN	CLOSE	402	1095	508	1469
Gullo	2020	-0.01659	0.032345	OPEN	CLOSE	555	790	560	784
Cobo	2010	-0.08262	0.020507	OPEN	FRESH	1877	3286	1976	3185
Sole	2013	-0.22317	0.043243	OPEN	FRESH	454	990	630	1099
Pujol	2019	0.049393	0.107844	OPEN	CLOSE	127	474	127	498
Buderatska	2020	0.02009	0.06531	OPEN	FRESH	90	121	191	262
De Gheselle	2020	-0.25328	0.067449	OPEN	FRESH	160	287	158	220
Cobo	2008	-0.22898	0.076264	OPEN	FRESH	125	231	149	219
Paffoni a	2011	-0.57966	0.088398	OPEN	FRESH	112	268	97	130
Paffoni b	2011	-1.04026	0.114695	CLOSE	FRESH	69	261	101	135
Papatheodorou	2013	-0.03493	0.043885	OPEN	CLOSE	378	608	385	598
Garcia	2011	-0.2754	0.046285	CLOSE	FRESH	184	283	596	696
Trokoudes	2011	-0.1173	0.050191	OPEN	FRESH	155	210	205	247
Papatheodorou	2016	-0.06775	0.027635	CLOSE	FRESH	692	984	739	982
Gallardo	2016	0.030539	0.130214	CLOSE	FRESH	43	68	46	75
Parmegiani	2011	-0.34954	0.1089	OPEN	FRESH	76	168	77	120
Forman	2012	-0.44015	0.053549	OPEN	FRESH	170	294	264	294
Antinori	2007	-0.05399	0.021023	OPEN	FRESH	295	330	685	726
Montjean D	2015	-0.35042	0.05348	OPEN	FRESH	295	648	349	540

Supplementary Table S4: Treatment effect and standard error per study for the cleavage rate per 2PN zygote outcome.

AUTHOR	YEAR	Treatment Effect	Standard Error of Treatment Effect	Group 1	Group 2	Cleaved embryos group 1	2PN zygotes group 1	Cleaved embryos group 2	2PN zygotes group 2
De Munck	2016	0.062217	0.071168	OPEN	CLOSE	133	188	117	176
Rienzi	2010	-0.02116	0.014966	OPEN	FRESH	93	95	100	100
Porcu	2021	-0.00238	0.020648	OPEN	CLOSE	402	445	508	561
Gullo	2020	0.002747	0.015496	OPEN	CLOSE	555	594	560	601
Cobo	2010	-0.01741	0.012928	OPEN	FRESH	1877	2256	1976	2334
Sole	2013	-0.03956	0.034041	OPEN	FRESH	454	665	630	887
Pujol	2019	-0.08887	0.092893	OPEN	CLOSE	127	294	127	269
Buderatska	2020	0.007058	0.044223	OPEN	FRESH	90	102	191	218
De Gheselle	2020	0	0.00628	OPEN	FRESH	160	160	158	158
Cobo	2008	-0.12434	0.057514	OPEN	FRESH	125	171	149	180
Paffoni a	2011	-0.01468	0.022719	OPEN	FRESH	112	116	97	99
Paffoni b	2011	-0.18348	0.058869	CLOSE	FRESH	69	87	101	106
Papatheodorou	2013	-0.01099	0.018299	OPEN	CLOSE	378	406	385	409
Garcia	2011	-0.0174	0.015241	CLOSE	FRESH	184	191	596	608
Trokoudes	2011	-0.00121	0.021998	OPEN	FRESH	155	162	205	214
Papatheodorou	2016	0.001846	0.014068	CLOSE	FRESH	692	744	739	796
Gallardo	2016	0.026085	0.086775	CLOSE	FRESH	43	51	46	56
Parmegiani	2011	-0.02246	0.08582	OPEN	FRESH	76	107	77	106
Forman	2012	-0.08776	0.023733	OPEN	FRESH	170	187	264	266
Antinori	2007	-0.00882	0.012103	OPEN	FRESH	295	305	685	702
Montjean D	2015	-0.2369	0.027324	OPEN	FRESH	295	376	349	351

Supplementary Table S5: Treatment effect and standard error per study for the blastocyst formation rate per oocyte retrieved outcome.

AUTHOR	YEAR	Treatment Effect	Standard Error of Treatment Effect	Group 1	Group 2	Blastocysts group 1	MII oocytes retrieved group 1	Blastocysts group 2	MII oocytes retrieved group 2
Gullo	2020	-0.05429	0.060687	OPEN	CLOSE	314	790	329	784
Buderatska	2020	-0.11199	0.099977	OPEN	FRESH	64	121	155	262
De Gheselle	2020	-0.53779	0.139642	OPEN	FRESH	64	287	84	220
Garcia	2011	-0.35105	0.106353	CLOSE	FRESH	79	283	276	696

Chamayou	2017	-0.26192	0.104249	OPEN	FRESH	138	615	135	463
Papatheodorou	2016	-0.09734	0.048003	CLOSE	FRESH	440	984	484	982
Forman	2012	-0.73089	0.126449	OPEN	FRESH	65	294	135	294
Chang	2013	-0.00448	0.148511	OPEN	FRESH	59	186	65	204
Montjean D	2015	-0.86599	0.158103	OPEN	FRESH	53	648	105	540

Supplementary Table S6: Treatment effect and standard error per study for the blastocyst formation rate per 2PN zygote outcome.

AUTHOR	YEAR	Treatment Effect	Standard Error of Treatment Effect	Group 1	Group 2	Blastocysts group 1	2PN zygotes group 1	Blastocysts group 2	2PN zygotes group 2
Gullo	2020	-0.03495	0.053636	OPEN	CLOSE	314	594	329	601
Buderatska	2020	-0.12502	0.087667	OPEN	FRESH	64	102	155	218
De Gheselle	2020	-0.28451	0.122273	OPEN	FRESH	64	160	84	158
Garcia	2011	-0.09305	0.096959	CLOSE	FRESH	79	191	276	608
Chamayou	2017	-0.15888	0.096464	OPEN	FRESH	138	411	135	343
Papatheodorou	2016	-0.02775	0.041695	CLOSE	FRESH	440	744	484	796
Forman	2012	-0.3785	0.116983	OPEN	FRESH	65	187	135	266
Chang	2013	0.113307	0.133166	OPEN	FRESH	59	124	65	153
Montjean D	2015	-0.75247	0.151272	OPEN	FRESH	53	376	105	351

Supplementary Table S7: Treatment effect and standard error per study for the clinical pregnancy rate outcome.

AUTHOR	YEAR	Treatment Effect	Standard Error of Treatment Effect	Group 1	Group 2	Clinical Pregnancies group 1	Patients group 1	Clinical Pregnancies group 2	Patients group 2
De Munck	2016	0.182322	0.499019	OPEN	CLOSE	6	17	5	17
Rienzi	20	-0.14953	0.2283	OPEN	FRESH	15	40	54	124

	10								
Porcu	2021	-0.18714	0.133256	OPEN	CLOSE	79	369	95	368
Gullo	2020	-0.04349	0.149821	OPEN	CLOSE	45	95	47	95
Cobo	2010	0.027399	0.083872	OPEN	FRESH	148	300	144	300
Sole	2013	0.120144	0.141218	OPEN	FRESH	53	99	47	99
Pujol	2019	0.223144	0.25	OPEN	CLOSE	20	40	16	40
Papatheodorou	2013	-0.25131	0.24081	OPEN	CLOSE	21	75	27	75
Garcia	2011	0.028988	0.161401	CLOSE	FRESH	21	34	51	85
Trokoudes	2011	0.130053	0.218705	OPEN	FRESH	20	36	20	41
Papatheodorou	2016	0.057158	0.128013	CLOSE	FRESH	54	92	51	92
